# Computed Tomography Angiography as a Method for Diagnosing Intracavitary Coronary Arteries

**DOI:** 10.3390/diagnostics14161798

**Published:** 2024-08-16

**Authors:** Paweł Gać, Bartosz Siudek, Agnieszka Głuszczyk, Jakub Plizga, Filip Grajnert, Rafał Poręba

**Affiliations:** 1 Centre of Diagnostic Imaging, 4th Military Hospital, Weigla 5, 50-981 Wrocław, Poland; 2 Department of Population Health, Division of Environmental Health and Occupational Medicine, Wroclaw Medical University, Mikulicza-Radeckiego 7, 50-345 Wrocław, Poland; 3 Department of Angiology and Internal Diseases, Wroclaw Medical University, Borowska 213, 50-556 Wrocław, Poland

**Keywords:** coronary computed tomography angiography, intracavitary coronary arteries, left anterior descending, right coronary artery

## Abstract

The intracavitary coronary arteries (ICCA) course is a rare phenomenon, where the segments of the coronary artery go through the atria or ventricles of the heart. In the past, these changes were incidentally detected during invasive diagnostic procedures for other reasons, as well as during postmortem examinations. As the use of multidetector computed tomography angiography (CTA) becomes more widespread, it has emerged that the incidence of ICCA has been underestimated. We present images from two coronary computed tomography angiography cases, which document the existence of ICCA in patients with non-specific chest pain. In the first case, in a 66-year-old woman, in addition to confirming coronary artery disease without significant stenosis (CAD-RADS 2-category 2 in the coronary-artery-disease-reporting and data system), the course of the middle section of the right coronary artery (RCA) in the lumen of the right atrium was demonstrated. In the second case, in a 47-year-old man in whom the presence of atherosclerotic lesions in the coronary arteries was excluded (CAD-RADS 0), the course of the distal segment of the left anterior descending (LAD) was found in the lumen of the apical layers of the right ventricle. To sum up, it should be stated that coronary CTA is a non-invasive diagnostic method that allows for visualization of the ICCA. In coronary CTA performed for indications consistent with the guidelines of scientific societies, attention should also be paid to the possible intracavitary course of the coronary arteries. The identification of such a course of the coronary arteries may be useful when preparing the patient for potential future invasive procedures involving the cardiac cavities.

**Figure 1:**
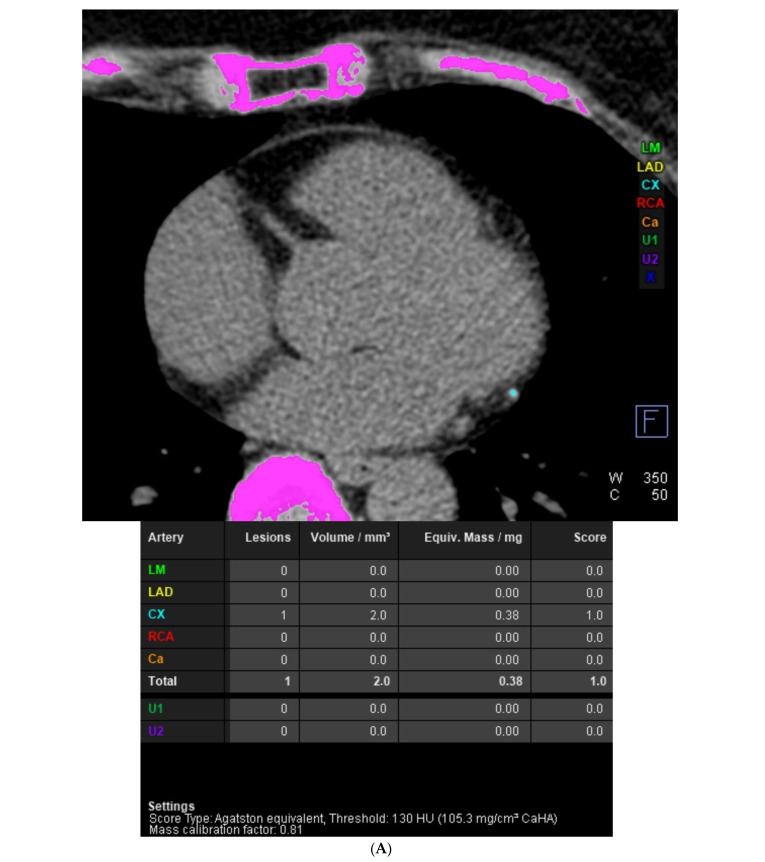

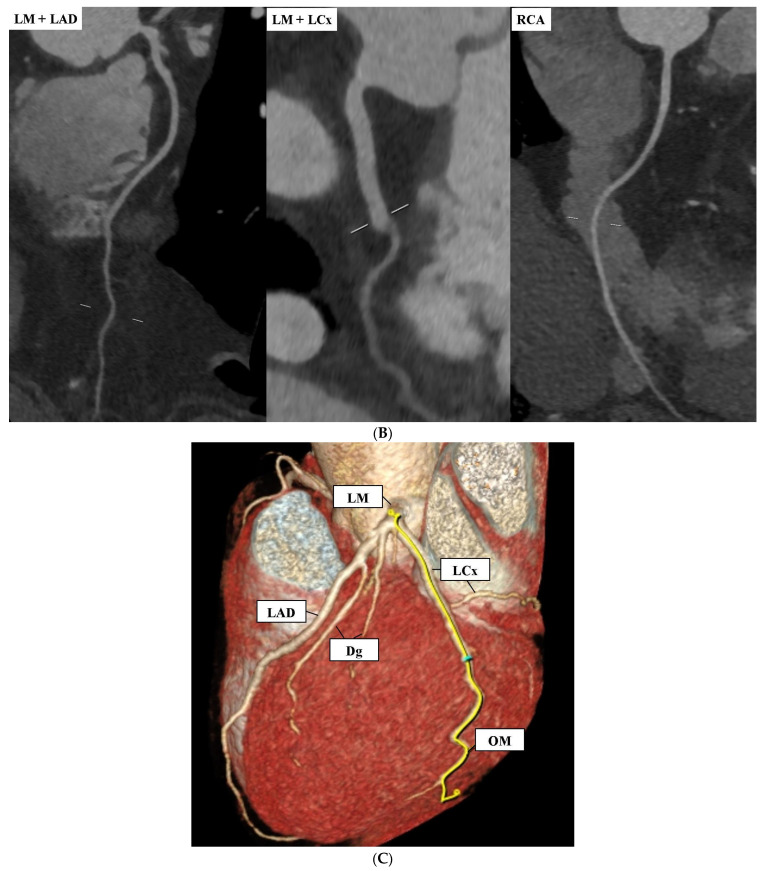

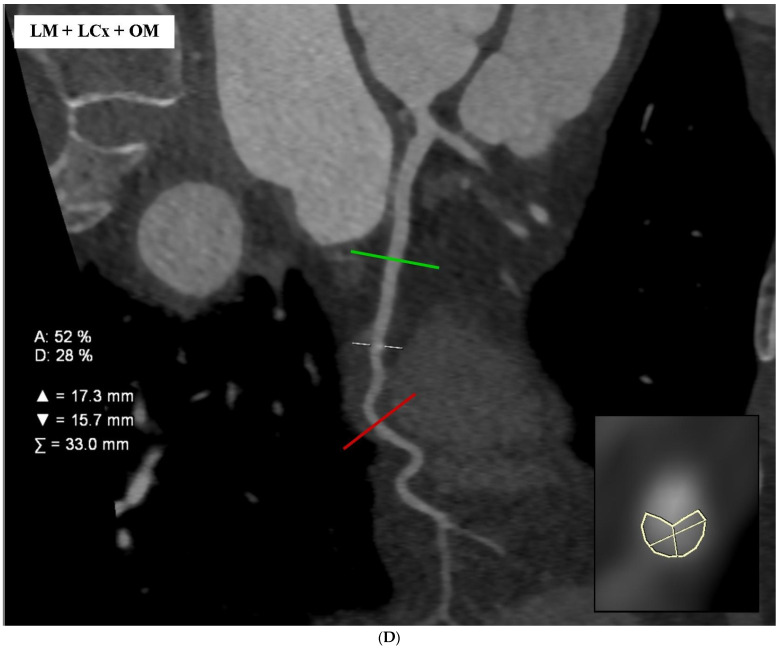

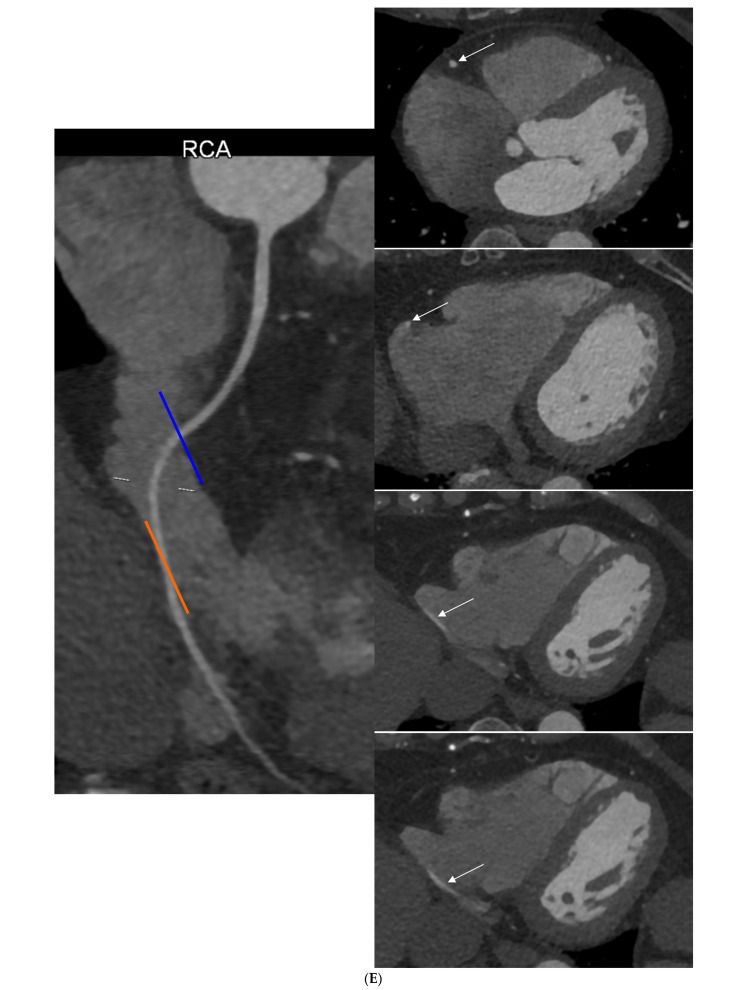

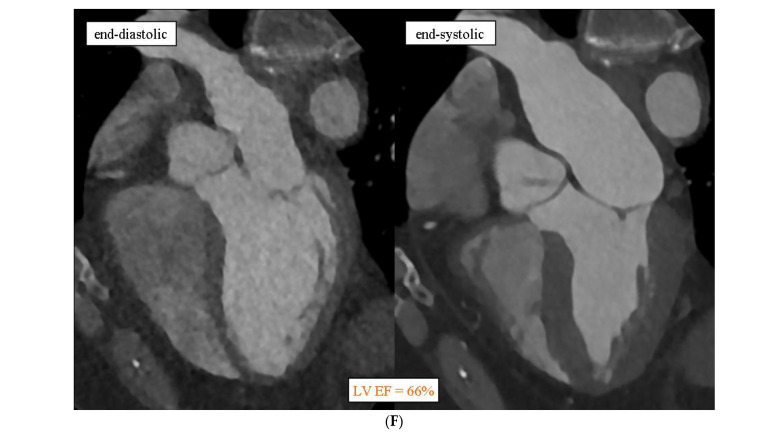
(**A**) Case 1: Intra-atrial course of the right coronary artery (RCA) in coronary computed tomography angiography. This study was performed using a 384-slice, 2-source Siemens Somatom Force computed tomography scanner (Siemens Healthcare, Erlangen, Germany). A typical coronary CTA protocol was used, including a native phase dedicated to the assessment of the coronary artery calcium score (CACS) and an angiographic phase after the administration of an iodinated contrast agent dedicated to the assessment of the coronary arteries and the morphology and function of the heart chambers. In the first case, in a 66-year-old woman, in addition to confirming coronary artery disease without significant stenosis (CAD-RADS 2), the course of the middle section of the right coronary artery (RCA) in the lumen of the right atrium was demonstrated. (**A**) CACS is 1.0 and results from the presence of one calcified lesion in the branches of the left circumflex artery (LCx). In the image, the calcified anatomical structures automatically recognized by the post-processing application are marked in pink. Calcifications assigned to the left main (LM) would be indicated in green, to the left anterior descending (LAD) in yellow, to the right coronary artery (RCA) in red, to other branches of the coronary arteries (CA) in orange, and to extracoronary structures (U1 and U2) with dark green and violet colors. Structures incorrectly identified as calcifications would be marked in dark blue. The calcified atherosclerotic plaque in the LCx branches is shown in blue. (**B**) The left main (LM), left anterior descending (LAD), and LCx are patent, with no visible atherosclerotic lesions and no stenoses. The LCx distal to the obtuse marginal branch (OM) is clearly recessive. RCA is dominant, patent, without atherosclerotic changes, and without stenosis. The image shows cMPR (multi-plane, curved) reconstructions of coronary arteries. The next image panels from the left show the reconstruction of the LM branch becoming the LAD, the reconstruction of the LM branch becoming the LCx, and the reconstruction of the RCA. (**C**) The image documents the left coronary circulation on VRT (volume rendering) reconstruction. LM, LAD, LCx, diagonal branches (Dg), and OM are marked. The yellow centerline runs through LM and the proximal sections of LCx and OM, after which the distal section of LCx is recessive. (**D**) The well-developed OM branch is patent. In the middle section of the OM, the calcified atherosclerotic plaque is visible, causing insignificant stenosis (25–50%). In summary, the case was classified as CAD-RADS 2. The image shows a cMPR (multiplanar, curved) reconstruction, including the LM, LCx, and OM. The green line indicates the reference level of the artery above stenosis, the red line indicates the reference level of the artery below stenosis, and the white dashed line indicates the level of arterial stenosis. In the lower left corner, the additional panel shows a cross-section perpendicular to the long axis of the artery at the level of stenosis. The white outline shows the contrasted lumen of the artery at the level of stenosis, and the lines within the outline show the maximum and minimum dimensions of the arterial lumen. (**E**) Itias revealed that the middle segment of the RCA, approximately 3.5 cm in length, runs in the lumen of the right atrium. The image shows the intra-atrial course of the RCA. The left panel shows a cMPR reconstruction of the right coronary artery, the blue line indicates the beginning of the RCA course in the right atrium lumen, the white dashed line indicates the center of the RCA course in the right atrium lumen, and the orange line indicates the end of the RCA course in the right atrium. The next panels on the right show axial images. White arrows indicate successive RCA segments in these panels. From the top, these will be the level above the intra-atrial RCA segment, the beginning of the intra-atrial RCA segment, the middle of the intra-atrial RCA segment, and the end of the intra-atrial RCA segment. (**F**) Left ventricular systolic function is preserved. The estimated left ventricular ejection fraction (LV EF) is 66%. The image shows long-axis, four-chamber images from different phases of the cardiac cycle. On the left panel, there is an end-diastolic image; on the right panel, there is an end-systolic image. The frequency of intracavitary coronary arteries (ICCA) course based on computed tomography scans was estimated in previous studies to range from 0.36% [1] to 1.3% [2]. In a cohort study, where the frequency of this anomaly was found to be 1.3%, the most common anomaly was associated with the left anterior descending artery (LAD) at 0.9%, followed by the right coronary artery (RCA) at 0.4%. Due to the increased number of implanted pacemakers and other invasive cardiac procedures performed, attention should be paid to the potential risk of coronary artery injury. Uncommon anomalies of the right coronary artery (RCA) are not frequent, and abnormal courses of the RCA are even rarer. There is only one paper in which a systematic review of case reports about the intra-atrial course of the RCA was performed. The authors noted that most cases with intra-atrial RCA course involved women, and patients were aged between 45 and 78 years [3,4,5]. Regarding the frequency of intra-atrial course of specific coronary artery segments, the following observations were made: the mid-segment of the RCA was the most common segment to have an intra-atrial course, representing 76.2% of the studied group; the distal segment of the RCA accounted for 23.8% of these cases; none of these patients showed an intra-atrial course involving the proximal RCA segment; and there were no cases where the artery exhibited an intra-atrial course in two segments simultaneously [3]. Regarding the lengths of the intra-atrial course of the coronary artery, data were collected on the lengths of the artery and the specific area of the RCA segment located intramuscularly. The distance of the mid-segment ranged from 13.2 mm to 55 mm. As for the extent of the distal segment, it ranged from 14 mm to 53 mm [5]. Interestingly, in none of the cited cases were coronary artery stenoses detected, nor were signs of atherosclerosis found there. The authors explain this situation via the lack of mechanical stress on the segments of the arteries that course intramurally, rather than physiologically, in the epicardial fat tissue [6].

**Figure 2:**
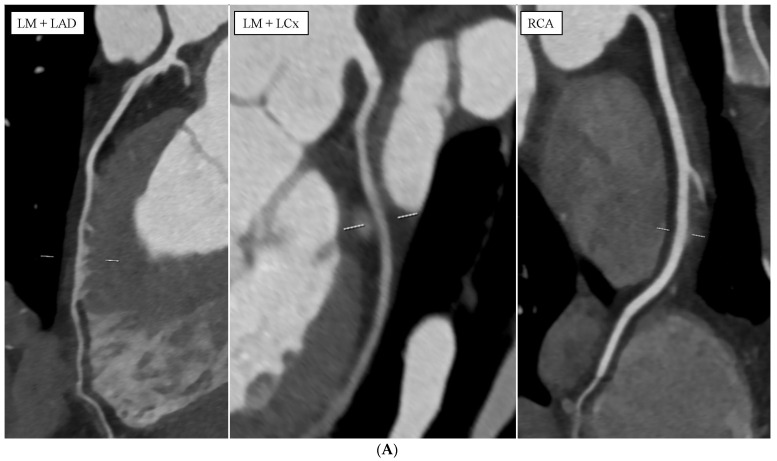

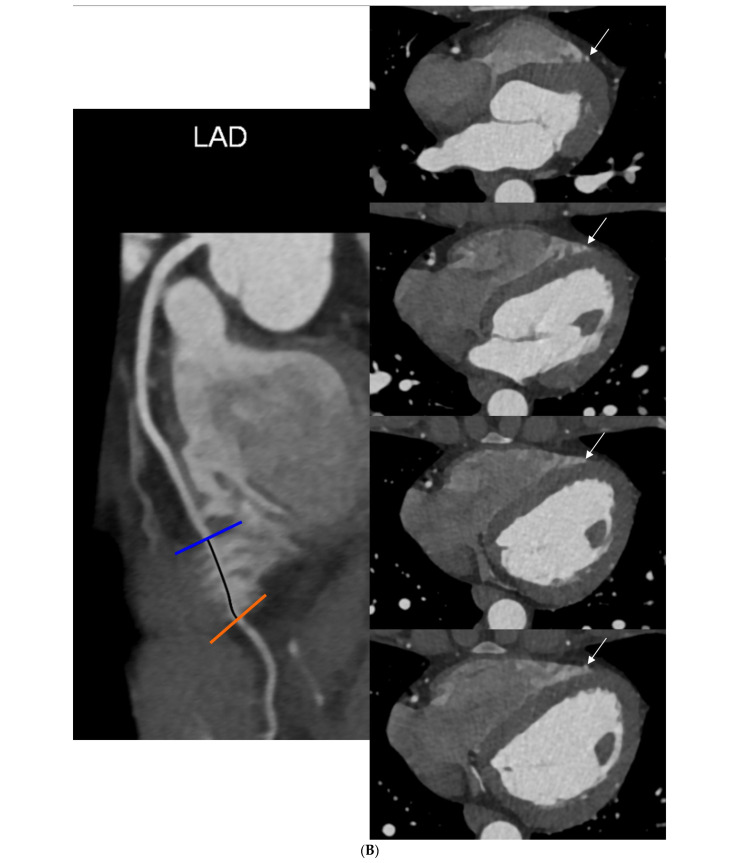

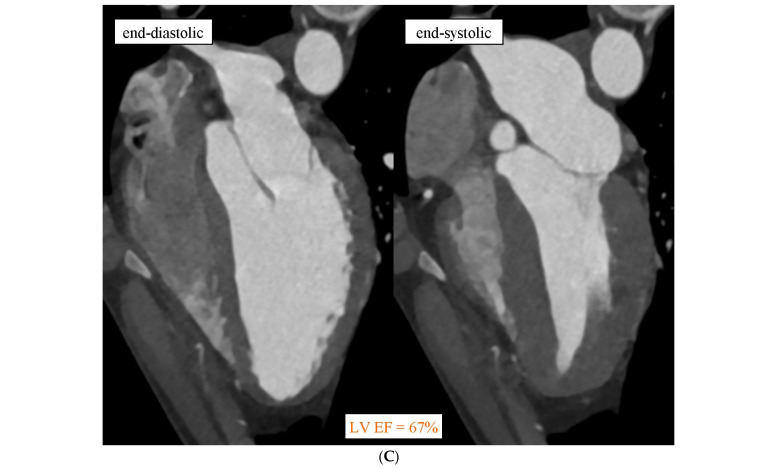
(**A**) Case 2: Intraventricular course of the left anterior descending (LAD) in coronary computed tomography angiography. This study was performed using a 384-slice, two-source Siemens Somatom Force computed tomography scanner (Siemens Healthcare, Erlangen, Germany). A typical coronary CTA protocol was used, including a native phase dedicated to the assessment of the coronary artery calcium score (CACS) and an angiographic phase after the administration of an iodinated contrast agent dedicated to the assessment of the coronary arteries and the morphology and function of the heart chambers. In the second case, in a 47-year-old man in whom the presence of atherosclerotic lesions in the coronary arteries was excluded (CAD-RADS 0), the course of the distal segment of the left anterior descending (LAD) was found in the lumen of the apical layers of the right ventricle. CACS was 0, indicating no population risk of significant coronary artery disease. (**A**) LM, LAD, well-developed diagonal branches (Dg), LCx, and the dominant RCA are patent, without atherosclerotic lesions and without stenoses. The image shows reconstructions of cMPR. In the following panels, from the left: LM becoming LAD; LM becoming LCx and RCA. (**B**) It is shown that in the distal section of the LAD, for a length of approximately 1.8 cm, it runs in the lumen of the apical layers of the right ventricle. The image shows the intraventricular course of the LAD. The left panel shows a cMPR reconstruction of the LAD, the blue line indicates the beginning of the LAD course in the right ventricle lumen, the white centerline indicates the entire intraventricular LAD segment within the right ventricular lumen, and the orange line indicates the end of the LAD course in the right ventricle. The next panels on the right show axial images. White arrows indicate successive LAD segments in these panels. From the top, these will be the level above the intraventricular LAD segment, the beginning of the intraventricular LAD segment, the middle of the intraventricular LAD segment, and the end of the intraventricular LAD segment. (**C**) Left ventricular systolic function is also preserved. The estimated left ventricular ejection fraction was 67%. The image shows long-axis, four-chamber images from different phases of the cardiac cycle. On the left panel, there is an end-diastolic image; on the right panel, there is an end-systolic image. The intraventricular (IV) LAD usually enters the right ventricle (RV) early in the descending course, enters the muscular layer at an acute angle, and exits to the surface at a shallower angle in a long curve. If a sudden change in the depth of the course of this artery is noticed, IV or intramyocardial rupture may be suspected with high probability [7]. In a retrospective study by Tyczyński et al., 31,748 pieces of coronary angiography data from 2008–2019 were summarized. In 17 patients, the intraventricular course of coronary arteries was demonstrated. All the arteries in the right ventricle (RV) were LAD. The segment running in the RV was located on the border between the free wall of the RV and the interventricular septum. It was shown that this course concerned the middle and distal segments of the LAD. It was also revealed that the average distance from the entry of the coronary artery to the RV in these subjects was 74.5 ± 17.1 mm. The average length of the intraventricular artery was 25.1 ± 14.0 mm [8]. A study conducted by La Mura et al., which included patients who underwent the examination from 2021 to 2022, showed that 14 (1.73%) of the subjects had an intraventricular course of the coronary artery. In 11 of them, this course concerned LAD running in the RV [9]. When describing the case by Hussein et al., they drew attention to possible underestimations of this phenomenon. This may result from the increasingly widespread use of coronary computed tomography angiography [10]. In research conducted by Buckley et al., 39 of patients with intraventricular LAD were identified, where the average length of the intraventricular course was 15.6 mm [1].

Typically, patients with intracavitary coronary arteries (ICCA) course did not present symptoms, and the detection of this type of anomaly occurred during coronary CTA conducted due to other symptoms such as palpitations, atrial fibrillation [11], atypical chest pain [12], chest tightness [13], dyspnea on exertion, chest pain, nausea, diaphoresis [14], hypertension, or previous acute coronary syndrome [1]. It has also been observed that individuals with an intramural course of the right coronary artery (RCA) more commonly exhibit other anomalies related to coronary arteries. Additionally, some patients have described an intramuscular course of the left anterior descending artery (LAD) or non-physiologic origins of the left circumflex artery [6]. Diagnosed ICCA usually does not require any special therapeutic approach. However, traumatic injury to the coronary artery with an intracavitary course during other medical procedures usually requires cardiac surgery [15].

To sum up, it should be stated that CCTA is a non-invasive diagnostic method with high sensitivity and specificity in ICCA imaging. Therefore, in CCTA examinations performed for indications consistent with the guidelines of scientific societies, attention should also be paid to the possible intracavitary course of the coronary arteries. The identification of such a course of the coronary arteries may be useful when preparing the patient for potential future invasive procedures involving the heart chambers.
